# Predicting hemodynamics in native and residual coarctation: preliminary results of a Rigid-Wall Computational-Fluid-Dynamics model (RW-CFD) validated against clinically invasive pressure measures at rest and during pharmacological stress

**DOI:** 10.1186/1532-429X-13-S1-P49

**Published:** 2011-02-02

**Authors:** Israel Valverde, Cristina Staicu, Heynric Grotenhuis, Alberto Marzo, Kawal Rhode, Yubing Shi, Alistair G Brown, Aphrodite Tzifa, Tarique Hussain, Gerald Greil, Patricia Lawford, Reza Razavi, Rod Hose, Philipp Beerbaum

**Affiliations:** 1 Kings College London, London, UK; 2 Department of Cardiovascular Science, Medical Physics Group. University of Sheffield, Sheffield, UK; 3 Leiden University Medical Centre, Leiden, Netherlands

## Introduction

Current clinical assessment of borderline aortic coarctation may involve cardiovascular magnetic resonance(CMR) but if inconclusive, invasive catheterization pressure measurements are required to evaluate the pressure gradient at rest and during pharmacological stress(isoprenaline).

## Purpose

To predict the aortic pressure distribution in patients with aortic coarctation at rest and pharmacological stress using a transient rigid-walled computation fluid dynamics model(RW-CFD).

## Methods

The study cohort comprises 5 patients with native or recurrent aortic coarctation and 2 control patients with healthy aortic arches(Table 1), who underwent both CMR(1.5-Intera,Philips) and catheterization at rest and pharmacological stress.

**Table 1:** Study population demographics

Study number	Age [years]	Weight [kg]	Body surface area [m^2^]	Clinical condition	Previous procedures
**Aortic coarctation**					

AoCo-1	15	59	1.7	Residual aortic coarctation, mitral stenosis	Coarctation repair (end-to-end anastomosis)
AoCo-2	25	64	1.8	Residual aortic coarctation, atrial septal defect	Coarctation repair (end-to-end anastomosis) and ASD repair
AoCo-3	21	95	2.1	Residual aortic coarctation, bicuspid aortic valve	Coarctation repair (Dacron patch)
AoCo-4	20	71	1.9	Residual aortic coarctation	Coarctation repair (subclavian flap)
AoCo-5	17	71	1.9	Native aortic coarctation	None

**Normal aorta**					

1	2	15	0.6	Partial anomalous pulmonary venous return, residual pulmonary vein stenosis	PAPVR repair
2	1	8.4	0.4	Biliar artesia	None

**Figure 1:**
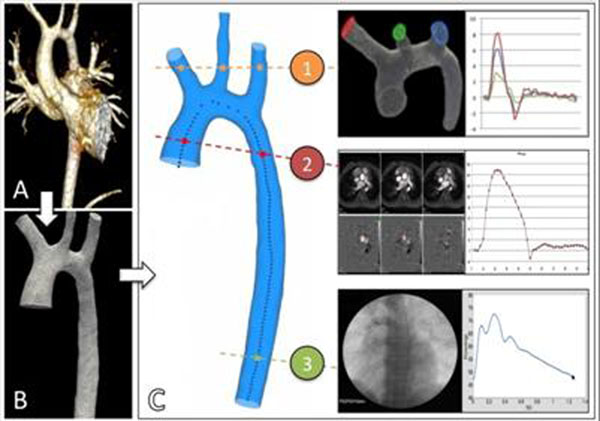
Workflow to run the CFD simulation. A. Contrast enhanced cardiovascular magnetic resonance (CMR) acquired to image the aortic arch. B. Extracted aortic geometry from the CMR dataset. C. Boundary condition setup in the three openings of the aortic geometry: 1) The applied flow rate is constructed as a proportion of the inlet flow in order to have a constant wall shear stress [l/min] 2) Phase-contrast CMR flow obtained in the ascending aorta [l/min]. 3) Catheter pressure measurements at the level of the diaphragmatic aorta [mmHg].

The model workflow(Figure 1) requires,as input parameters,the aortic geometry, extracted from the CMR 3D gadolinium contrast-enhanced sequence(TR=4.4ms,TE=1.8ms,1.5x1.5x1.8mm), and definition of the boundary conditions. The blood flow(modelled as a Newtonian incompressible fluid) in the aortic domain is conditioned by the clinical data at three locations: ***1)Ascending aortic root****:* The inlet flow is extracted from the phase-contrast CMR flow(TR=4.7ms,TE=3ms,2.5x2.5x7mm,80 phases). **2) *Supra-aortic vessels***:The flow rate is calculated as a proportion of the inlet flow based on the assumption of a constant wall shear stress (*Kundu,2004*).**3) *Diaphragmatic aorta***: The pressure waveform is extracted from the invasive catheter investigation.

The clinically invasive aortic pressure gradients were compared with the predicted pressure distribution along the centreline in the RW-CFD model at the time of peak flow(Table 2).

**Table 2:** Study results in patients with aortic coarctation (AoCo) at rest and stress conditions

Study number	Heart rate [bpm]	Cardiac Output [l/min/m^2^]	Pressure Ascending Aorta Invasive [mmHg]	Pressure Diaphragmatic Aorta Invasive [mmHg]	ΔP Clinical Invasive [mmHg]	ΔP CFD [mmHg]	Pressure Difference (CFD – Invasive) [mmHg]
**Rest condition**							

AoCo-1	48	2.0	102 ± 3	79	23 ± 3	22	**-1 ± 3**
AoCo-2	86	3.2	82 ± 3	64	18 ± 3	5	**-13 ± 3**
AoCo-3	69	2.2	86 ± 4	74	12 ± 4	9	**-2 ± 4**
AoCo-4	81	2.8	78 ± 2	68	10 ± 2	8	**-2 ± 2**
AoCo-5	47	1.9	82 ± 2	73	9 ± 2	6	**-3 ± 2**

**Stress**							

AoCo-1	150	5.6	116 ± 6	77	39 ± 6	54	**18 ± 6**
AoCo-2	136	5.4	103 ± 10	63	40 ± 10	23	**-17 ± 10**
AoCo-3	130	5.6	121 ± 6	57	64 ± 6	42	**-22 ± 6**
AoCo-4	140	6.5	152 ± 4	86	66 ± 4	44	**-22 ± 4**
AoCo-5	141	7.2	114 ± 7	77	37 ± 7	58	**21 ± 7**

## Results

For patients with aortic coarctation, during pharmacological stress, there was an increase in both heart rate(72±21bpm,*mean±standard deviation*) and invasive pressure gradient drop across the coarctation(35±18mmHg,Table 2). The RW-CFD model predicted accurately the pressure drop at rest (-4.2±4.9 mmHg), and moderate agreement at stress (-4.4±21.9 mmHg. Table 2, Figure 3).

For healthy controls, the RW-CFD model predicted the absence of a significant gradient both at rest and stress(1±1mmHg) .

**Figure 2:**
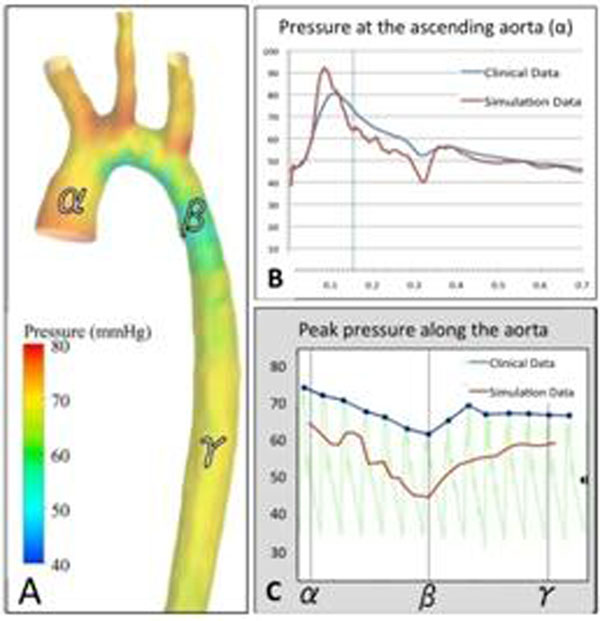
CFD model results (AoCo-5) A. CFD model pressure distribution at the instant of peak flow (vertical line) along the aorta: ascending aorta (α), aortic concentration (β) and diaphragmatic aorta (γ). B. Pressure curve comparison over one cardiac cycle at the level of the ascending aorta (α), assessed by clinical catheterization invasive measurements (blue line) and simulated by the CFD model (red line). C. Peak pressure distribution comparison across the aortic centerline from the ascending (α) to the diaphragmatic aorta (γ), assessed by invasive catheter pullback (blue line) and simulated by the CFD model (red line). Note the pressure drop at the level of coarctation (β) and posterior recovery. [Pressure expressed in mmHg].

**Figure 3:**
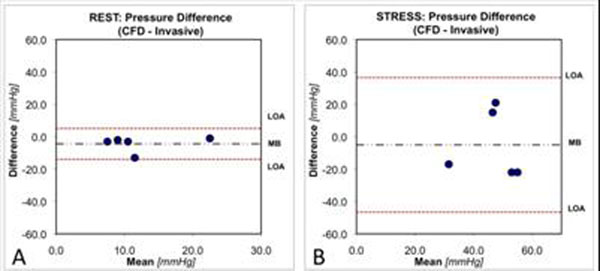
Bland-Altman plots: Comparison of the clinically invasive and the predicted CFD pressure gradient (mmHg) across the aortic coarctation at rest (A) and isoprenaline pharmacological stress (B). The dot-dashed grey horizontal lines represent the mean difference between CFD and invasive data (MB, mean bias), and the paired dotted red horizontal lines represent ±2 standard deviations from this mean difference (LOA), 95% limits of agreement). Note the increased bias during the stress condition compared to the good agreement at rest condition.

## Conclusion

For patients with aortic coarctation,the RW-CFD simulations accurately predict the pressure gradient at rest and give indication of the gradient severity during stress.Furthermore, no gradient was predicted in control patients with normal aortae.

These preliminary results, whilst using a simple CFD approach and a small cohort of patients, are quite promising. This study represents the first step towards an image-based fluid-solid-interaction CFD analysis. This more sophisticated approach is likely to overcome the current limitations and might grant additional information.

In the future, it is envisaged that CFD models could be based on a patient-specific, non-invasive and non-ionising radiation assessment such as CMR in order to predict the hemodynamic conditions in the aorta and avoid invasive cardiac catheterization.

The pressures assessed by clinically invasive catheterization at the level of the ascending aorta, diaphragmatic aorta and the pressure gradient across the coarctation (ΔP Clinical Invasive) are compared with the RW-CFD model pressure gradient prediction (ΔP CFD). The absolute pressure differences values are shown in the final column. Pressures expressed as mean ± standard deviation. bpm, beats per minute.

